# Increased risk of second primary malignancies among endometrial cancer survivors receiving surgery alone: A population‐based analysis

**DOI:** 10.1002/cam4.3861

**Published:** 2021-09-15

**Authors:** Yen‐Ling Lai, Chun‐Ju Chiang, Yu‐Li Chen, San‐Lin You, Yun‐Yuan Chen, Ying‐Cheng Chiang, Yi‐Jou Tai, Heng‐Cheng Hsu, Chi‐An Chen, Wen‐Fang Cheng

**Affiliations:** ^1^ Department of Obstetrics and Gynecology National Taiwan University Taipei Taiwan; ^2^ Department of Obstetrics and Gynecology National Taiwan University Hospital Hsin‐Chu City Taiwan; ^3^ Graduate Institute of Epidemiology and Preventive Medicine College of Public Health National Taiwan University Taipei Taiwan; ^4^ Taiwan Cancer Registry Taipei Taiwan; ^5^ Department of Public Health College of Medicine and Big Data Research Centre Fu‐Jen Catholic University New Taipei City Taiwan; ^6^ Taiwan Blood Services Foundation Taipei Taiwan; ^7^ Graduate Institute of Clinical Medicine National Taiwan University Taipei Taiwan; ^8^ Graduate Institute of Oncology College of Medicine National Taiwan University Taipei Taiwan

**Keywords:** endometrial cancer, genetic testing, lynch syndrome, second primary cancer, standardized incidence ratio

## Abstract

**Background:**

Women with endometrial cancer (EC) have favorable prognoses, leaving them vulnerable to the development of second primary cancers (SPCs). We investigated the SPC risk and survival outcomes among EC patients treated with surgery alone in order to exclude the impact of adjuvant treatment on the results.

**Methods:**

Data from the Taiwan Cancer Registry from 1995 to 2013 were analyzed. Standardized incidence ratios (SIRs) of SPCs among EC survivors were calculated.

**Results:**

Among 7725 women enrolled, 478 developed an SPC. The overall SIR for SPCs in EC survivors was 2.84 (95% confidence interval [CI] 2.59–3.10) compared with the general female population. Women diagnosed with EC at age <50 years had a higher SIR for an SPC than those diagnosed at age ≥50 years (SIR = 4.38 vs. 1.28). The most frequent site of an SPC was the small intestine (SIR = 8.39, 95% CI 2.72–19.58), followed by the kidney (SIR = 4.84, 95% CI 1.78–10.54), and oral cavity (SIR = 4.52, 95% CI 2.17–8.31). Women, regardless of age at EC diagnosis, had significantly higher SIRs for subsequent breast, colorectal, lung, and thyroid cancer, and lymphoma. Women with an SPC had shorter overall survival than those without (5‐year: 88.9 vs. 94.2%, 10‐year: 71.3 vs. 89.8%, 15‐year: 62.3 vs. 86.1%, and 20‐year: 47.6 vs. 81.1%, all ps<0.001).

**Conclusions:**

Even women treated for EC with surgery alone, especially young EC survivors, had an increased risk of SPCs. Genetic counseling/testing is recommended for young EC patients, and all are recommended to receive regular surveillance and screening for breast, colorectal, and lung cancers.

## 1 INTRODUCTION

Endometrial cancer (EC) is the most common cancer of the female reproductive tract and one of the few cancers with increasing incidence and mortality.^1^, ^2^, ^3^ In the United States, uterine corpus cancer incidence rates increased 0.7% per year from 1999 to 2015, and mortality rates increased 1.1% per year from 1999 to 2016.^2^ This trend has also been observed in Asian countries.^4^, ^5^, ^6^ In Taiwan, the age‐adjusted incidence rate of endometrioid EC increased from 0.83 per 100,000 women per year between 1979 and 1983 to 7.50 per 100,000 women per year between 2004 and 2008.^6^ It is noteworthy that Asian women were younger at diagnosis of EC and had better outcomes compared with Western patients.^5^, ^7^

Cancer survivors are at increased risk of developing subsequent cancers compared with the general population.^8^ As EC survivors have relatively long‐term survival, information about the risk of second primary cancers (SPCs) following EC is essential, and there is an urgent need for evidence‐based guidelines for routine surveillance and screening for subsequent malignancies among EC patients. The risk of an SPC might be increased due to shared genetic and/or environmental risk factors common to both cancers. For example, Lynch syndrome is an autosomal dominant hereditary disorder associated with an increased probability of developing certain types of cancer, including EC and colorectal cancer,^9^ and SPC can be a late effect of treatment modalities administered for primary cancer, especially radiotherapy.^10^, ^11^, ^12^, ^13^, ^14^, ^15^ Postoperative radiotherapy is considered among EC patients with selected pathologic risk factors. Numerous studies have reported increased risks of SPCs among women with EC, and have emphasized the role of radiotherapy in the underlying mechanism of subsequent cancers after EC.^16^, ^17^, ^18^, ^19^, ^20^, ^21^

To our knowledge, there is no study that has evaluated the risk of an SPC among the EC patients who received surgery without any adjuvant treatment. The objective of the present study was to explore the probability of developing an SPC, the prevalences of specific cancer types, and the survival outcomes of the EC patients treated with surgery alone.

## 2 MATERIALS AND METHODS

### 2.1 Data collection

**FIGURE 1:**
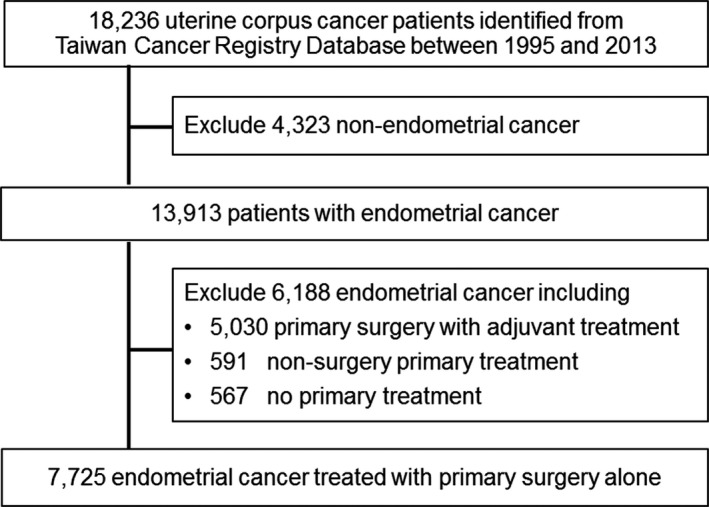
Flowchart of cohort selection of this study. (Adjuvant treatment included chemotherapy, radiotherapy, chemoradiotherapy, immunotherapy, hormonal therapy, and targeted therapy)

This study was approved by the National Taiwan University Hospital Research Ethics Committee. A total of 18,236 women with the diagnosis of primary uterine corpus cancer (ICD‐O, 182 or C54) were retrieved from the Taiwan Cancer Registry (TCR) database from January 1, 1995 to December 31, 2013. The disease codes were based on the International Classification of Diseases for Oncology (ICD‐O). Since 2002, ICD‐O‐3 instead of ICD‐O‐FT has been used for coding the site and the histology of the neoplasm, usually obtained from a pathology report. Therefore, all cancer types before 2002 were converted to ICD‐O‐3 codes for the analysis. The flowchart of the cohort selection of this study is shown in Figure 1. The exclusion criteria included patients identified with non‐EC uterine corpus cancer (*n* = 4323), patients with EC who received primary surgery followed by adjuvant treatment (*n *= 5030), patients with EC who received nonsurgical primary treatment (*n *= 591), and patients with EC who did not receive primary treatment (*n* = 567). Primary treatment was defined as the treatment administered to patients within 12 months after the initial diagnosis. In addition, patients who did not receive chemotherapy, radiotherapy, chemoradiotherapy, hormonal therapy, or targeted therapy within 12 months after diagnosis of EC were defined as not having adjuvant treatment. Finally, the remaining 7725 patients diagnosed with EC who received primary surgery alone were eligible for this study. We also identified which of these 7725 patients were diagnosed with SPCs in different organs or tissues after the diagnosis of EC. We followed the SPC occurrences for all EC cases until December 31, 2015. Patients diagnosed with subsequent cancers within 2 months of the initial EC diagnosis were excluded.

The TCR is a population‐based cancer registry founded by the Ministry of Health and Welfare of the central government, Taiwan in 1979. All newly diagnosed malignant neoplasms from hospitals with greater than 50‐bed capacity have been reported to the registry. Passive follow‐up was conducted using the national death certificate database maintained by the Department of Statistics, Ministry of Health and Welfare, Taiwan. The duration of survival for each case was determined as the time from the date of initial diagnosis to the date of death, or date of follow‐up termination (December 31, 2015), whichever came first.

### 2.2 Statistical analysis

Standardized incidence ratios (SIRs) and the corresponding 95% confidence intervals (CIs) of SPCs among EC survivors were analyzed to quantify the relative risk compared with cancers among women in the general female population by the exact method.^22^ The SIR was calculated as the ratio of the observed number of cases in the study cohort to the expected number of cases in the reference population, assuming the patients in the study cohort demonstrated cancer rates equivalent to those for individuals in the general female population. The expected number of cases was computed using age‐specific rates from a reference population, weighted according to the age structure of the study population. The number of person‐years at risk (PYRs) was defined as the number of years from the date of diagnosis of EC to the date of death, date of SPC diagnosis, or the date of follow‐up termination, whichever occurred first. The PYRs and observed cases of cancer were stratified according to 5‐year age groups and calendar year. Cancer incidence rates were computed for each second cancer site according to age and calendar year and were multiplied by the accumulated PYRs to estimate the expected number of subsequent cancers for each stratum. The SIRs of SPCs of EC women were estimated by age at EC diagnosis (<50 years or ≥50 years), and its confidence intervals were based on the assumed Poisson distribution of second primary cancer cases. Additionally, cumulative incidence analysis was performed to estimate the cumulative risk of SPCs. Kaplan–Meier survival curves were calculated for all EC patients with or without an SPC and stratified by age at EC diagnosis (<50 years or ≥50 years). Differences between the two age groups were assessed using a log‐rank test. Two‐tailed *p* values <0.05 were considered statistically significant. All statistical analyses were conducted using SAS software 9.4 version (SAS^®^ Institute Inc., Cary, NC).

## 3 RESULTS

### 3.1 Patient characteristics

A total of 7725 women with an initial diagnosis of EC treated with primary surgery alone without any adjuvant treatment including chemotherapy, radiotherapy, chemoradiotherapy, immunotherapy, targeted therapy, and hormone therapy were recruited in this study. As shown in Table 1, the median follow‐up period was 82 months. The median age at initial diagnosis of EC was 52 years, and 2962 (38.3%) women were diagnosed with EC when younger than 50 years old. There were 478 (6.19%) women who developed an SPC during 61,173 person‐years of follow‐up, including 119 diagnosed with EC at age <50 years and 359 diagnosed with EC at age ≥50 years. The median age at diagnosis of the SPC was 61 years and the median interval from the initial diagnosis of EC to the diagnosis of the second cancer was 64 months.

**TABLE 1:** Characteristics of 7725 endometrial cancer patients treated with primary surgery alone in Taiwan between 1995 and 2013

	All	Age <50 years	Age ≥50 years
Patients with endometrial cancer (%)	7725 (100%)	2962 (38.3%)	4763 (61.7%)
Year of endometrial cancer diagnosis			
1995–1999	863	385	478
2000–2004	1520	656	864
2005–2009	2542	978	1564
2010–2013	2800	943	1857
Age at diagnosis of endometrial cancer, median (range, years)	52 (18–93)	44 (18–49)	57 (50–93)
Patients with a second primary cancer (%)	478 (100%)	119 (24.9%)	359 (75.1%)
Age at diagnosis of second primary cancer, median (range, years)	61 (31–94)	51 (31–64)	65 (50–94)
Median interval between endometrial cancer and second primary cancer (range, months)	64 (2–239)	75 (3–239)	63 (2–233)
Median follow‐up (range, months)	82 (0–251)	95 (1–251)	75 (0–251)

### 3.2 Risk of second primary cancers classified by location

SIRs and the corresponding 95% CIs for SPCs at different anatomical sites were further calculated. As shown in Table 2, the overall SIR for an SPC in EC survivors was 2.84 (95% CI 2.59–3.10). There was a significantly increased risk of cancers of the following sites/types: small intestine (SIR = 8.39, 95% CI 2.72–19.58), kidney (renal cell carcinoma, RCC) (SIR = 4.84, 95% CI 1.78–10.54), oral cavity and pharynx (SIR = 4.52, 95% CI 2.17–8.31), lymphoma (SIR = 4.32, 95% CI 2.71–6.54), renal pelvis (transitional cell carcinoma, TCC) (SIR = 4.18, 95% CI 2.16–7.30), colorectal (SIR = 4.18, 95% CI 3.39–5.10), lung (SIR = 3.94, 95% CI 3.03–5.04), thyroid gland (SIR = 3.55, 95% CI 2.32–5.20), pancreas (SIR = 3.43, 95% CI 1.65–6.31), leukemia (SIR = 3.33, 95% CI 1.66–5.97), breast (SIR = 3.15, 95% CI 2.60–3.79), liver (SIR = 2.51, 95% CI 1.77–3.44), stomach (SIR = 2.34, 95% CI 1.34–3.81), and skin (SIR = 2.11, 95% CI 1.09–3.69) cancers (Table 2). Among the colorectal cancers, the SIR was highest in the ascending colon (SIR = 7.91, 95% CI 5.41–11.17) followed by the transverse colon (SIR = 3.85, 95% CI 1.76–7.30), and descending colon (including rectum) (SIR = 3.35, 95% CI 2.49–4.40). With respect to the different histologic types of lung cancer, lung adenocarcinoma was the most common histologic type, accounting for 73% (46/63) of all second primary lung cancers.

**TABLE 2:** Risk of second primary cancers after diagnosis of endometrial cancer with primary surgery alone in Taiwan between1995 and 2013

Location	All (*N* = 7725)		Age <50 years (*N* = 2962)		Age ≥50 years (*N* = 4763)	
	SIR (O/E)	95%CI	SIR (O/E)	95%CI	SIR (O/E)	95%CI
Oral cavity & pharynx	**4.52** (10/2.21)	2.17–8.31	5.99 (2/0.33)	0.73–21.63	2.12 (8/3.77)	0.92–4.18
Major salivary gland	4.35 (2/0.46)	0.53–15.73	—	—	3.57 (2/0.56)	0.43–12.90
Nasopharynx	1.45 (3/2.01)	0.30–4.24	—	—	1.34 (3/2.25)	0.28–3.90
Stomach	**2.34** (16/6.83)	1.34–3.81	2.82 (2/0.71)	0.34–10.17	1.08 (14/12.94)	0.59–1.82
Small intestine	**8.39** (5/0.60)	2.72–19.58	**64**.**79** (4/0.06)	17.65–165.88	0.88 (1/1.13)	0.02–4.93
Colorectum	**4.18** (97/23.20)	3.39–5.10	**14**.**39** (29/2.02)	9.64–20.67	**1.49** (68/45.61)	1.16–1.80
A‐colon	**7.91** (32/4.04)	5.41–11.17	**34**.**07** (8/0.23)	14.71–67.13	**2.85** (24/8.43)	1.82–4.23
T‐colon	**3.85** (9/2.34)	1.76–7.30	**18**.**60** (4/0.22)	5.07–47.61	1.10 (5/4.55)	0.36–2.56
D‐colon and rectum	**3.35** (51/15.22)	2.49–4.40	**9.74** (14/1.44)	5.32–16.34	1.26 (37/29.46)	0.88–1.73
Anus	8.15 (2/0.25)	0.99–29.45	—	—	4.14 (2/0.48)	0.50–14.96
Unspecified site	2.23 (3/1.34)	0.46–6.53	**28**.**24** (3/0.11)	5.82–82.52	—	—
Liver	**2.51** (38/15.17)	1.77–3.44	**8.61** (6/0.70)	3.16–18.74	0.99 (32/32.37)	0.68–1.40
Gallbladder	2.11(4/1.89)	0.58–5.41	**36**.**98** (3/0.08)	7.63–108.08	0.25 (1/4.06)	0.01–1.37
Pancreas	**3.43** (10/2.91)	1.65–6.31	**18**.**59** (3/0.16)	3.83–54.32	1.15 (7/6.10)	0.46–2.36
Nasal cavity	3.45 (1/0.29)	0.09–19.21	—	—	2.35 (1/0.43)	0.06–13.07
Lung	**3.94** (63/15.99)	3.03–5.04	**7.91** (9/1.14)	3.62–15.02	**1.66** (54/32.46)	1.25–2.17
Adenocarcinoma	**4.15** (46/11.07)	3.04–5.54	**8.75** (5/0.57)	2.84–20.41	**2.27** (36/14.86)	1.39–3.14
Small cell carcinoma	4.42 (2/0.45)	0.53–15.95	—	—	2.41 (2/0.83)	0.29–8.72
SCC	3.11 (4/1.29)	0.85–7.97	—	—	2.48 (6/2.42)	0.91–5.40
Breast	**3.15** (115/36.47)	2.60–3.79	**4.38** (31/9.36)	3.63–5.24	**1.82** (84/46.23)	1.45–2.25
Vagina/vulva	3.98 (3/0.75)	0.82–11.65	—	—	2.28 (3/1.32)	0.47–6.65
Kidney (RCC)	**4.84**(6/1.24)	1.78–10.54	**12**.**64** (2/0.16)	1.53–45.64	1.80 (4/2.23)	0.49–4.60
Renal pelvis (TCC)	**4.18**(12/2.87)	2.16–7.30	**22**.**68**(2/0.09)	2.75–81.93	1.58 (10/6.31)	0.76–2.91
Bladder	2.09 (6/2.87)	0.77–4.56	7.61 (1/0.13)	0.19–42.41	0.82 (5/6.12)	0.27–1.91
Brain	2.82 (4/1.42)	0.77–7.21	2.34 (1/0.43)	0.06–13.02	1.95 (3/1.54)	0.40–5.69
Thyroid gland	**3.55** (26/7.33)	2.32–5.20	**4.82** (12/2.49)	2.49–8.41	**2.07** (14/6.77)	1.13–3.47
Skin	**2.11** (12/5.68)	1.09–3.69	**6.20** (3/0.48)	1.28–18.11	0.80 (9/11.21)	0.37–1.52
Leukemia	**3.33 (11/3.30)**	1.66–5.97	2.41 (2/0.83)	0.29–8.72	2.12 (9/4.25)	0.97–4.02
Lymphoma	**4.32 (22/5.09)**	2.71–6.54	**5.01** (4/0.80)	1.36–12.82	**2.11** (18/8.54)	1.25–3.33
Total	**2.84** (478/168.55)	2.59–3.10	**4.38** (119/27.16)	3.63–5.24	**1.28** (359/279.89)	1.15–1.42

Bold type indicates statistical significance.

Abbreviations: CI, confidence interval; O/E, ratio of the observed number of second primary cancers to the number of expected cancers; RCC, renal cell carcinoma; SCC, squamous cell carcinoma; SIR, standardized incidence ratio; TCC, transitional cell carcinoma.

### 3.3 Risk of second primary cancers classified by age at diagnosis of EC

We further evaluated the risk of SPCs in different age groups. As shown in Table 2, women diagnosed with EC at age <50 years had a higher risk of developing an SPC than those diagnosed at age ≥50 years (SIR = 4.38 vs. 1.28). There were six types of cancer that showed significantly higher SIRs in women diagnosed with EC at age <50 years, but not in those diagnosed at age ≥50 years, including those of the small intestine (SIR = 64.79, 95% CI 17.65–165.88), kidney (SIR = 12.64, 95% CI 1.53–45.64), renal pelvis (SIR = 22.68, 95% CI 2.75–81.93), liver (SIR = 8.61, 95% CI 3.16–18.74), pancreas (SIR = 18.59, 95% CI 3.83–54.32), and skin (SIR = 6.20, 95% CI 1.28–18.11) (Table 2). There were five types of cancer, including colorectal cancer, lung cancer, breast cancer, thyroid cancer, and lymphoma, that showed significantly higher SIRs regardless of age. The SIRs of these five types were higher in EC patients diagnosed at age <50 years than in EC patients diagnosed at age ≥50 years. In the analysis stratified by the subsites of colorectal cancer, the SIRs of ascending colon cancer were higher in EC patients diagnosed at age <50 years than in EC patients diagnosed at age ≥50 years (SIR = 34.07 vs. 2.85). The SIRs of transverse and descending colon cancers were only significantly higher in women diagnosed with EC at age <50 years. Similarly, the SIR of lung adenocarcinoma was also higher in EC patients diagnosed at age <50 years than those diagnosed at age ≥50 years (SIR = 8.23 vs. 1.75).

### 3.4 Cumulative incidence rates of second primary cancers in EC patients

**FIGURE 2:**
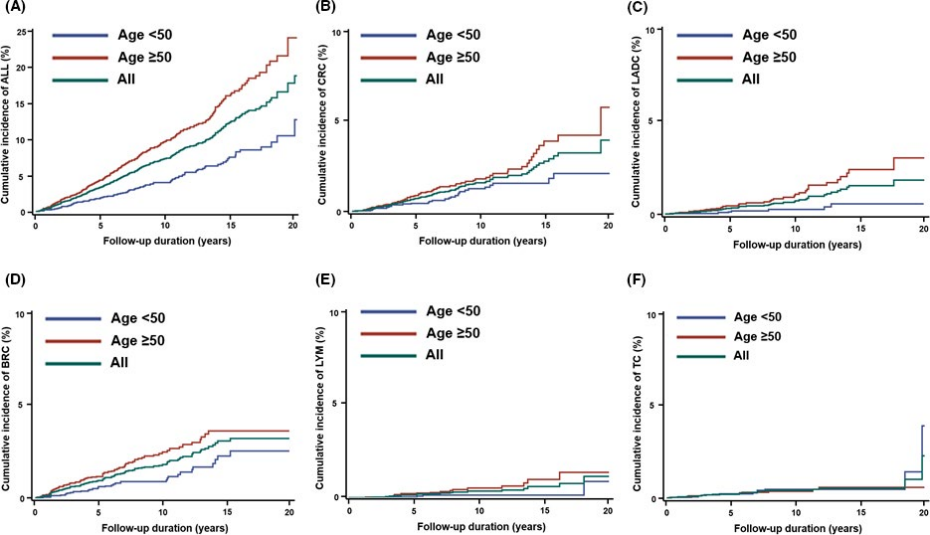
Cumulative incidences of EC patients with respective second primary cancer. (A) All second primary cancers, (B) colorectal cancer, (C) lung adenocarcinoma, (D) breast cancer, (E) lymphoma, (F) thyroid cancer

The estimated risks of developing SPCs in EC survivors treated with primary surgery alone were further evaluated. As shown in Table 3, the overall cumulative incidence rates of all second primary cancers at 5, 10, 15, and 20 years were 3.3%, 7.2%, 12.2%, and 18.7%, respectively. The cumulative incidence rates were higher in EC patients diagnosed at age ≥50 years than in EC patients diagnosed at age <50 years (4.3% vs. 1.9%, 9.6% vs. 4.0%, 15.9% vs. 7.4%, and 23.9% vs. 12.6% at 5, 10, 15, and 20 years, respectively; all *p*<0.001; Figure 2A). When focusing on five types of cancers with significantly higher SIRs in both age groups, four cancers (all but thyroid cancer) had significantly higher cumulative incidence rates among EC patients diagnosed at age ≥50 years, including colorectal cancer (Figure 2B), lung adenocarcinoma (Figure 2C), breast cancer (Figure 2D), and lymphoma (Figure 2E), compared with EC patients diagnosed at age <50 years (for 20 years of follow‐up, colorectal cancer 5.7 vs. 2.1%, lung adenocarcinoma 3.0% vs. 0.5%, breast cancer 3.6% vs. 2.5%, and lymphoma 1.3% vs. 0.8%; all *p*<0.05). The cumulative incidences of thyroid cancer were similar between the two age groups (Figure 2F).

**TABLE 3:** Cumulative incidence rates of second primary cancers of endometrial cancer patients after 5, 10, 15, and 20 years of follow‐up

	5 years			10 years			15 years			20 years		
	All	Age <50	Age ≥50	All	Age <50	Age ≥50	All	Age <50	Age ≥50	All	Age <50	Age ≥50
All second primary cancers	3.3	1.9	4.3	7.2	4.0	9.6	12.2	7.4	15.9	18.7	12.6	23.9
Major sites												
Colorectal cancer	0.7	0.4	0.9	1.6	1.2	1.8	2.6	1.5	3.6	3.9	2.1	5.7
Lung adenocarcinoma	0.3	0.1	0.4	0.6	0.2	0.9	1.5	0.5	2.4	1.8	0.5	3.0
Breast cancer	0.9	0.6	1.1	1.7	0.8	2.4	3.0	2.2	3.6	3.2	2.5	3.6
Lymphoma	0.1	0.0	0.2	0.3	0.1	0.5	0.6	0.1	1.0	1.1	0.8	1.3
Thyroid cancer	0.2	0.2	0.2	0.4	0.5	0.4	0.5	0.5	0.6	2.2	3.9	0.6

### 3.5 Overall survival rates of EC patients with or without second primary cancers

**FIGURE 3:**
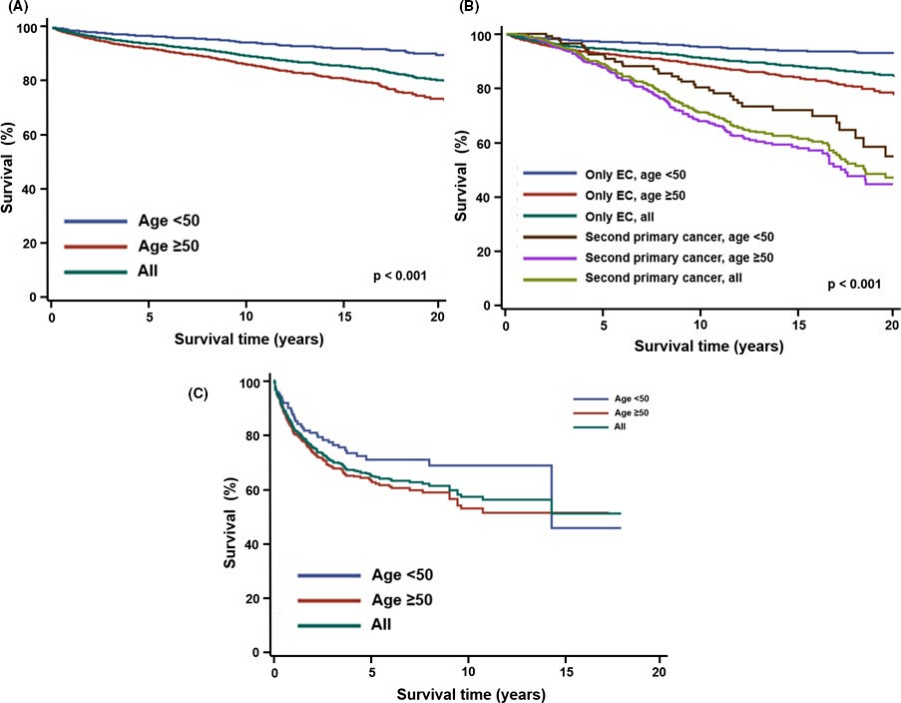
Overall survival rates of EC patients from the diagnosis of EC and from the diagnosis of second primary cancers stratified by age at diagnosis of EC (<50 or ≥50 years). (A) All EC patients, from the diagnosis of EC, (B) EC patients with or without second primary cancers, from the time of diagnosis of EC, (C) EC patients with second primary cancers, from the time of diagnosis of second primary cancers

The 5‐, 10‐, 15‐, and 20‐year overall survival (OS) rates of women after the diagnosis of EC were 94.2%, 89.8%, 86.1%, and 81.1%, respectively (Figure 3A). Women diagnosed with EC at age <50 years had significantly higher OS rates than those diagnosed at age ≥50 years (5‐year 97.0 vs. 92.4%, 10‐year 94.5 vs. 86.7%, both ps <0.001, Figure 3A). Among the women with SPCs after EC, the 5‐, 10‐, 15‐, and 20‐year OS rates were 88.9%, 71.3%, 62.3%, and 47.6%, respectively (Figure 3B). Among these women with SPCs, significantly higher OS rates were observed in patients diagnosed with EC at age <50 years than in patients diagnosed with EC at age ≥50 years (5‐year 92.4 vs. 87.7%, 10‐year 80.5 vs. 68.2%, *p* <0.001, Figure 3B). The OS rates of EC survivors with SPCs (5‐year 88.9, 10‐year 71.3%, 15‐year 62.3 and 20‐year 47.6 were significantly lower and declined sharply 5 years after the diagnosis of EC compared with survivors without SPCs (5‐year 94.2%, 10‐year 89.8%, 15‐year 86.1% and 20‐year 81.1%) (*p*<0.0001, Figure 3B).

### 3.6 Overall survival rates after the diagnosis of second primary cancers in EC survivors

Finally, we evaluated the survival of EC survivors after the diagnosis of SPC. The 5‐year and 10‐year OS rates of these women after the diagnosis of the SPCs were 65.4% and 57.1%, respectively (Figure 3C). The women diagnosed with EC at age <50 years (5‐year 70.9% and 10‐year 68.6%) had significantly higher OS rates after SPCs than those diagnosed with EC at age ≥50 years (5‐year 63.6% and 10‐year 52.7%) (*p* = 0.044, Figure 3C).

## 4 DISCUSSION

Asian women were younger at the diagnosis of endometrial cancer. The age of diagnosis for EC differed between Asian and western countries. Of the women in this study cohort, 38.3% was diagnosed at age <50 years. Several other investigators have reported results similar to ours.^6^, ^21^, ^23^, ^24^ However, less than 25% of EC is diagnosed before menopause in western countries.^7^, ^25^, ^26^ Asian women also have more favorable outcomes than non‐Asian women.^5^, ^7^ Molecular alterations involved in the pathogenesis of EC may contribute to racial differences.^27^ The incidence of p53 overexpression was higher in African Americans than in Caucasians, and *PTEN* mutations were fourfold more frequent in Caucasians relative to African Americans.^28^ These findings suggest that molecular alterations could be one explanation for racial differences in survival.^28^, ^29^, ^30^, ^31^ However, no comprehensive information of molecular alterations in Taiwanese patients with EC has been investigated. Further studies on detailed molecular alterations in EC and environmental risk factors are warranted to elucidate the etiology of racial disparities in the age of onset and survival.

Women with EC have a greater tendency to develop subsequent cancers than does the general female population. The overall risk of developing SPCs among all women diagnosed with EC was higher than in the general female population, especially in women younger than 50 years old in our series and in several other studies.^16^, ^17^, ^18^, ^19^, ^20^, ^21^ Two analyses using the SEER database showed that elevated overall risk for a second primary cancer among all EC survivors was limited to women less than 50 years old and black women.^15^, ^16^ Further investigations are recommended to evaluate whether race and other confounding factors, including genetic and environmental factors, influence the development of SPCs among EC survivors.

EC survivors had higher risks of developing several types of SPCs. The small intestine was the most common site of cancer subsequent to treatment for EC in our series and other investigators’ studies.^16^, ^19^, ^21^, ^32^ The increased probability of developing small intestine cancers was limited to women diagnosed with EC at age <50 years in our study and the other two studies.^16^, ^21^ Inherited susceptibility syndromes including Lynch syndrome probably contribute to the elevated risk of small intestine cancer.^9^

Young women who had survived EC and were less than 50 years old had increased risks of developing SPCs in many organs. In addition to small intestine cancer mentioned above, subsequent kidney, renal pelvis, pancreas, liver, skin, transverse colon, and descending colon cancer also occurred only among women diagnosed with EC at age <50 years in this study. Other studies found results similar to ours for some organs, especially the colon and rectum.^17^, ^18^, ^21^ Young EC survivors’ higher risks for specific cancer types may be due to genetic predisposition. Predisposition to the early onset of certain types of primary cancers may be caused by germline mutations in DNA mismatch repair (MMR) genes or deletions in the *EPCAM* gene.^33^, ^34^ Win et al. estimated the risks of subsequent cancers following an initial EC for women carrying MMR gene mutations, and they observed an increased risk for a wide range of cancers, including colorectal cancer, kidney cancer, renal pelvis cancer, ureter cancer, urinary bladder cancer, and breast cancer compared with the general population.^9^ The prevalence of Lynch syndrome in Taiwanese women has not been estimated. However, this genetic association can provide insight into the increased risk of specific malignancies in young EC survivors. Diagnosis of Lynch syndrome is based on a combination of clinical phenotype, routine tumor pathology, and/or genetic screening practices.^35^ Therefore, we recommend genetic counseling for women diagnosed with EC at age <50 years or who have a family history of genetic diseases like Lynch syndrome. If a woman with EC is identified as having Lynch syndrome, screening strategies including annual colonoscopy and urine cytology coupled with ultrasound should be initiated.^35^

Women treated for EC had increased risks of developing subsequent breast, colorectum, lung, lymphoma, and thyroid gland cancers regardless of their age at diagnosis (<50 or ≥50 years). Colorectal cancer occurred more commonly among women diagnosed with EC at age <50 years. In addition, the risk of ascending colon cancer was highest followed by transverse colon cancer and descending colon cancer. Women who were ≥50 years old at EC diagnosis had an increased risk of developing colorectal cancer only of the ascending colon, not the transverse or descending colon, in our series. Our results are inconsistent with the previous studies. Some investigators did not observe an elevated risk of colorectal cancer among women treated for EC.^36^, ^37^ We speculate that the discrepant results may be due to the different study methodologies. The majority of previous studies used cancer registry databases without a control group. Therefore, they had limited ability to adjust potential confounding variables such as nonsteroidal anti‐inflammatory drug therapy, diabetes mellitus, body mass index, hormonal replacement therapy, oral contraceptive use, and family history of colorectal cancer. One limitation of this study is that we also obtained information from a population‐based cancer registry database, and thus were also unable to adjust for such confounding factors.

Several potential mechanisms may contribute to the increased risk of colorectal cancer among EC survivors, including hereditary association and multiple metabolic syndromes, especially diabetes and obesity, which are also prevalent in EC survivors.^38^ Demb et al. investigated the risk factors for colorectal cancer by anatomic site and found that diabetes prevalence is most significantly associated with increased risk of ascending colon cancer, compared with distal or rectal cancer.^39^ Obesity was associated with increased risk of colorectal cancer, regardless of site.^40^, ^41^, ^42^

Lung cancer, especially lung adenocarcinoma, and breast cancer were another two common SPCs among women treated for EC, especially in younger EC survivors. EC and breast cancer share some risk factors, such as nulliparity and exposure to estrogen.^43^, ^44^ Furthermore, Cowden syndrome and Lynch syndrome are genetic disorders considered to be involved in the pathogenesis of EC and breast cancer.^45^, ^46^ A Western lifestyle with a more highly caloric and higher fat diet, reduced fertility rates, and delayed childbearing has been associated with prolonged stimulation by endogenous estrogen.^47^ With regard to exogenous estrogen, higher daily intake of certain industrial environmental pollutants with estrogenic effects has been reported in Taiwan.^48^ These findings can potentially contribute to EC and increased risk of subsequent breast cancer. Although the risks of lung and thyroid cancer and lymphoma significantly increased among all EC survivors, no environmental risk factor or genetic association between EC and these cancers has been reported.

Radiation exposure is a known risk factor for cancer development. EC patients often receive adjuvant radiotherapy to the pelvis for local control. Several researchers reported that radiotherapy to the pelvis increases the risk of SPCs, particularly for cancers closer to the uterus, including colon, rectum and bladder cancers, and leukemia.^10^, ^11^, ^12^, ^13^, ^14^, ^15^, ^16^ In this study, we excluded EC patients who received adjuvant radiotherapy to avoid the influence of adjuvant irradiation in the EC survivors. Further study is mandatory to investigate the late effect of pelvic irradiation on the SPCs of primary EC survivors.

Younger women diagnosed with EC had better survival outcomes compared with older women. Pellerin et al. also reported that EC patients younger than 45 years of age had a lower incidence of advanced stage disease, a higher degree of tumor differentiation, and a better prognosis compared with patients older than 45 years.^49^ The survival outcome was poorer among EC patients with an SPC compared with EC patients without an SPC, particularly at 5 years after the diagnosis of EC. The reason for this delay may be that women with early mortality due to EC do not have a chance to develop an SPC.^18^ Another explanation is that younger women with EC have better performance status than older women. Truong et al. have shown that age and Karnofsky performance status are two significant predictors of overall survival in women with EC.^50^

The strength of this study is that our results were based on a high‐quality population‐based database from the TCR. Therefore, there was a low possibility of selection bias. This information is useful for the consultation about the survival outcomes and surveillance of EC patients. The limitation of this study is that it was a retrospective database study. Therefore, we do not have information on the lifestyles, family histories, or genetic risk factors of these women treated for EC. Another weakness is the limited information on the stage and histologic type of EC.

In conclusion, our results indicate that women treated for EC even with surgery alone are at increased risk of many kinds of SPCs possibly caused by genetic and environmental factors. Women diagnosed with EC at age <50 years had a higher probability of developing subsequent cancer after EC compared with women diagnosed with EC at age ≥50 years. Breast, colorectal, lung, and thyroid gland cancer and lymphoma were the five SPCs that developed in all populations regardless of age (<50 or ≥50) at diagnosis. Appropriate genetic counseling and testing are recommended for young women with EC. All women treated for EC are recommended to receive regular surveillance screening for breast, colorectal, and lung cancers.

## CONFLICTS OF INTERESTS

The authors declare no conflict of interest.

## ETHICS APPROVAL

This study was approved by the National Taiwan University Hospital Hsinchu Branch (Hsinchu, Taiwan) Research Ethics Committee (IRB: 109–012‐E). All the patients’ data were fully anonymized and the Research Ethics Committee waived the requirement for informed consent.

## Data Availability

The data that support the findings of this study are available on request from the corresponding author. The data are not publicly available due to privacy or ethical restrictions.

## References

[1] Cronin KA , Lake AJ , Scott S , et al. Annual report to the nation on the status of cancer, part I: national cancer statistics. Cancer. 2018;124:2785‐2800. 29786848 10.1002/cncr.31551 PMC6033186

[2] Henley SJ , Miller JW , Dowling NF , Benard VB , Richardson LC . Uterine cancer incidence and mortality—United States, 1999–2016. MMWR Morb Mortal Wkly Rep. 2018;67:1333‐1338. 30521505 10.15585/mmwr.mm6748a1 PMC6329484

[3] Lortet‐Tieulent J , Ferlay J , Bray F , Jemal A . International patterns and trends in endometrial cancer incidence, 1978–2013. J Natl Cancer Inst. 2018;110:354‐361. 29045681 10.1093/jnci/djx214

[4] Sorosky JI . Endometrial cancer. Obstet Gynecol. 2012;120:383‐397. 22825101 10.1097/AOG.0b013e3182605bf1

[5] Lin CH , Chen YC , Chiang CJ , et al. The emerging epidemic of estrogen‐related cancers in young women in a developing Asian country. Int J Cancer. 2012;130:2629‐2637. 21702035 10.1002/ijc.26249

[6] Huang CY , Chen CA , Chen YL , et al. Nationwide surveillance in uterine cancer: survival analysis and the importance of birth cohort: 30‐year population‐based registry in Taiwan. PLoS One. 2012;7:e51372. 23251510 10.1371/journal.pone.0051372 PMC3519542

[7] Mahdi H , Schlick CJ , Kowk LL , Moslemi‐Kebria M , Michener C . Endometrial cancer in Asian and American Indian/Alaskan Native women: tumor characteristics, treatment and outcome compared to non‐Hispanic white women. Gynecol Oncol. 2014;132:443‐449. 24316310 10.1016/j.ygyno.2013.11.028

[8] Liu L , de Vries E , Louwman M , et al. Prevalence of multiple malignancies in the Netherlands in 2007. Int J Cancer. 2011;128:1659‐1667. 20503267 10.1002/ijc.25480

[9] Win AK , Lindor NM , Winship I , et al. Risks of colorectal and other cancers after endometrial cancer for women with Lynch syndrome. J Natl Cancer Inst. 2013;105:274‐279. 23385444 10.1093/jnci/djs525 PMC3576323

[10] Boice JD , Engholm G , Kleinerman RA , et al. Radiation dose and second cancer risk in patients treated for cancer of the cervix. Radiat Res. 1988;116:3‐55. 3186929

[11] Curtis RE , Boice JD , Stovall M , et al. Relationship of leukemia risk to radiation dose following cancer of the uterine corpus. J Natl Cancer Inst. 1994;86:1315‐1324. 8064889 10.1093/jnci/86.17.1315

[12] Chaturvedi AK , Engels EA , Gilbert ES , et al. Second cancers among 104,760 survivors of cervical cancer: evaluation of long‐term risk. J Natl Cancer Inst. 2007;99:1634‐1643. 17971527 10.1093/jnci/djm201

[13] Kumar S , Shah JP , Bryant CS , et al. Second neoplasms in survivors of endometrial cancer: impact of radiation therapy. Gynecol Oncol. 2009;113:233‐239. 19249081 10.1016/j.ygyno.2008.12.039

[14] Lönn S , Gilbert ES , Ron E , Smith SA , Stovall M , Curtis RE . Comparison of second cancer risks from brachytherapy and external beam therapy after uterine corpus cancer. Cancer Epidemiol Biomarkers Prev. 2010;19:464‐474. 20142245 10.1158/1055-9965.EPI-09-0892 PMC2866968

[15] Felix AS , Linkov F , Maxwell GL , Ragin C , Taioli E . Racial disparities in risk of second primary cancers in endometrial cancer patients: analysis of SEER data. Int J Gynecol Cancer. 2011;21:309‐315. 21528021 10.1097/IGC.0b013e318206a098 PMC3081714

[16] Curtis RE , Freedman DM , Ron E , et al. In: Chapter 9 New Malignancies Following Cancer of the Uterine Corpus and Ovary: New Malignancies Among Cancer Survivors: SEER Cancer Registries, 1973‐2000. National Cancer Institute, NIH Publ. No. 05‐5302. Bethesda, MD 2006:231‐55.

[17] Singh H , Nugent Z , Demers A , Czaykowski PM , Mahmud SM . Risk of colorectal cancer after diagnosis of endometrial cancer: a population‐based study. J Clin Oncol. 2013;31:2010‐2015. 23569324 10.1200/JCO.2012.47.6481

[18] Lim MC , Won Y‐J , Lim J , et al. Second primary colorectal cancer among endometrial cancer survivor: shared etiology and treatment sequelae. J Cancer Res Clin Oncol. 2018;144:845‐854. 29445866 10.1007/s00432-018-2599-3 PMC5916981

[19] Hemminki K , Aaltonen L , Li X . Subsequent primary malignancies after endometrial carcinoma and ovarian carcinoma. Cancer. 2003;97:2432‐2439. 12733142 10.1002/cncr.11372

[20] Rhoades J , Vetter MH , Fisher JL , Cohn DE , Salani R , Felix AS . The association between histological subtype of a first primary endometrial cancer and second cancer risk. Int J Gynecol Cancer. 2019;29:290‐298. 30718311 10.1136/ijgc-2018-000014 PMC6504982

[21] Lee K‐D , Chen C‐Y , Huang H‐J , et al. Increased risk of second primary malignancies following uterine cancer: a population‐based study in Taiwan over a 30‐year period. BMC Cancer. 2015;15:393. 25957789 10.1186/s12885-015-1426-3 PMC4469104

[22] Brreslow N . Statistical Methods in Cancer Research . Vol. 2, The Design and Analysis of Cohort Studies. Geneva: World Health Organization; 1987. pp.69‐70.

[23] Heo J , Chun M , Oh YT , Noh OK . Psychiatric comorbidities among endometrial cancer survivors in South Korea: a nationwide population‐based, longitudinal study. J Gynecol Oncol. 2019;30:e15. 30740948 10.3802/jgo.2019.30.e15 PMC6393629

[24] Gao Y , Dai X , Lee AC , Wise NR , Shen F , Chen Q . Body mass index is negatively associated with endometrial cancer stage, regardless of subtype and menopausal status. J Cancer. 2018;9:4756‐4761. 30588261 10.7150/jca.21137 PMC6299378

[25] Gao Y , Zhao M , Dai X , Tong M , Wei J , Chen Q . The prevalence of endometrial cancer in pre‐ and postmenopausal Chinese women. Menopause. 2016;23:884‐887. 27272224 10.1097/GME.0000000000000684

[26] Zeleniuch‐Jacquotte A , Akhmedkhanov A , Kato I , et al. Postmenopausal endogenous oestrogens and risk of endometrial cancer: results of a prospective study. Br J Cancer. 2001;84:975‐981. 11286480 10.1054/bjoc.2001.1704 PMC2363831

[27] Yap OWS , Matthews RP . Racial and ethnic disparities in cancers of the uterine corpus. J Natl Med Assoc. 2006;98:1930‐1933. 17225836 PMC2569671

[28] Maxwell GL , Risinger JI , Hayes KA , et al. Racial disparity in the frequency of PTEN mutations, but not microsatellite instability, in advanced endometrial cancers. Clin Cancer Res. 2000;6:2999‐3005. 10955777

[29] Clifford SL , Kaminetsky CP , Cirisano FD , et al. Racial disparity in overexpression of the p53 tumor suppressor gene in stage I endometrial cancer. Am J Obstet Gynecol. 1997;176:S229‐S232. 9215213 10.1016/s0002-9378(97)70380-6

[30] Kohler MF , Carney P , Dodge R , et al. p53 overexpression in advanced‐stage endometrial adenocarcinoma. Am J Obstet Gynecol. 1996;175:1246‐1252. 8942496 10.1016/s0002-9378(96)70036-4

[31] Tarney CM , Tian C , Wang G , et al. Impact of age at diagnosis on racial disparities in endometrial cancer patients. Gynecol Oncol. 2018;149:12‐21. 28800945 10.1016/j.ygyno.2017.07.145 PMC6863162

[32] Brown AP , Neeley ES , Werner T , Soisson A , Burt RW , Gaffney DK . A population‐based study of subsequent primary malignancies after endometrial cancer: genetic, environmental, and treatment‐related associations. Int J Radiat Oncol Biol Phys. 2010;78:127‐135. 19910129 10.1016/j.ijrobp.2009.07.1692

[33] Vasen HF , Watson P , Mecklin JP , Lynch HT . New clinical criteria for hereditary nonpolyposis colorectal cancer (HNPCC, Lynch syndrome) proposed by the International Collaborative Group on HNPCC. Gastroenterology. 1999;116:1453‐1456. 10348829 10.1016/s0016-5085(99)70510-x

[34] Kempers MJE , Kuiper RP , Ockeloen CW , et al. Risk of colorectal and endometrial cancers in EPCAM deletion‐positive Lynch syndrome: a cohort study. Lancet Oncol. 2011;12:49‐55. 21145788 10.1016/S1470-2045(10)70265-5 PMC3670774

[35] Lynch HT , Snyder CL , Shaw TG , Heinen CD , Hitchins MP . Milestones of Lynch syndrome: 1895–2015. Nat Rev Cancer. 2015;15:181‐194. 25673086 10.1038/nrc3878

[36] Fornasarig M , Minisini A , Clementi S , et al. Risk analysis of colorectal cancer in women with endometrial carcinoma. Mol Med Rep. 2008;1:549‐553. 21479448

[37] Srinivasan R , Yang YX , Rubin SC , Morgan MA , Lewis JD . Risk of colorectal cancer in women with a prior diagnosis of gynecologic malignancy. J Clin Gastroenterol. 2007;41:291‐296. 17426469 10.1097/01.mcg.0000225587.85953.06

[38] Kurnit KC , Ward KK , McHale MT , Saenz CC , Plaxe SC . Increased prevalence of comorbid conditions in women with uterine cancer. Gynecol Oncol. 2015;138:731‐734. 26160712 10.1016/j.ygyno.2015.07.004

[39] Demb J , Earles A , Martínez ME , et al. Risk factors for colorectal cancer significantly vary by anatomic site. BMJ Open Gastroenterol. 2019;6:e000313. 10.1136/bmjgast-2019-000313 PMC6711437 31523441

[40] Dai Z , Xu YC , Niu L . Obesity and colorectal cancer risk: a meta‐analysis of cohort studies. World J Gastroenterol. 2007;13:4199‐4206. 17696248 10.3748/wjg.v13.i31.4199 PMC4250618

[41] Schlesinger S , Lieb W , Koch M , et al. Body weight gain and risk of colorectal cancer: a systematic review and meta‐analysis of observational studies. Obes Rev. 2015;16:607‐619. 25925734 10.1111/obr.12286

[42] Lauby‐Secretan B , Scoccianti C , Loomis D , Grosse Y , Bianchini F , Straif K . International agency for research on cancer handbook working group. Body fatness and cancer‐viewpoint of the IARC working group. N Engl J Med. 2016;375:794‐798. 27557308 10.1056/NEJMsr1606602 PMC6754861

[43] Brown SB , Hankinson SE . Endogenous estrogens and the risk of breast, endometrial, and ovarian cancers. Steroids. 2015;99(Pt A):8‐10. 25555473 10.1016/j.steroids.2014.12.013

[44] Eliassen AH , Hankinson SE . Endogenous hormone levels and risk of breast, endometrial and ovarian cancers: prospective studies. Adv Exp Med Biol. 2008;630:148‐165. 18637490

[45] Win AK , Lindor NM , Jenkins MA . Risk of breast cancer in lynch syndrome: a systematic review. Breast Cancer Res. 2013;15:R27. 23510156 10.1186/bcr3405 PMC3672741

[46] Tan MH , Mester JL , Ngeow J , Rybicki LA , Orloff MS , Eng C . Lifetime cancer risks in individuals with Germline PTEN mutations. Clin Cancer Res. 2012;18:400‐407. 22252256 10.1158/1078-0432.CCR-11-2283 PMC3261579

[47] Lee MM , Chang IY , Horng CF , Chang JS , Cheng SH , Huang A . Breast cancer and dietary factors in Taiwanese women. Cancer Causes Control. 2005;16:929‐937. 16132802 10.1007/s10552-005-4932-9

[48] Lu YY , Chen ML , Sung FC , Wang PS , Mao IF . Daily intake of 4‐nonylphenol in Taiwanese. Environ Int. 2007;33:903‐910. 17512594 10.1016/j.envint.2007.04.008

[49] Pellerin GP , Finan MA . Endometrial cancer in women 45 years of age or younger: a clinicopathological analysis. Am J Obstet Gynecol. 2005;193:1640‐1644. 16260203 10.1016/j.ajog.2005.05.003

[50] Truong PT , Kader HA , Lacy B , et al. The effects of age and comorbidity on treatment and outcomes in women with endometrial cancer. Am J Clin Oncol. 2005;28:157‐164. 15803010 10.1097/01.coc.0000143049.05090.12

